# Application of multigene panel testing for bleeding, thrombotic, and platelet disorders in patients and the general population in China

**DOI:** 10.1186/s43556-025-00283-6

**Published:** 2025-06-09

**Authors:** Yaohua Cai, Wenyi Lin, Jun Deng, Zhipeng Cheng, Yanyi Tao, Hui Lu, Yunqing Xia, Tingting Wu, Liang V. Tang, Yu Hu

**Affiliations:** 1 Institute of Hematology, Union Hospital, Tongji Medical College, Huazhong University of Science and Technology, No. 1277 Jiefang Avenue, Wuhan, Hubei 430022 China; 2 Key Laboratory of Biological Targeted Therapy, Ministry of Education, Huazhong University of Science and Technology, Wuhan, Hubei 430022 China; 3 Hubei Clinical and Research Center of Thrombosis and Hemostasis, Wuhan, Hubei 430022 China

**Keywords:** Next-generation sequencing, Genetic analysis, Pathogenic variants, Age of onset, Thrombosis, Coagulation factor

## Abstract

Bleeding, thrombotic, and platelet disorders (BTPDs) are rare but complex conditions with diverse clinical presentations that often delay accurate diagnosis. In this study, we developed an Expanded Thrombohemostasis (ExTH) gene panel comprising 130 diagnostic and risk-associated genes. This panel was applied to 747 patients and 760 controls, representing the largest genetic screening study for BTPDs in an Asian population to date. A high overall diagnostic yield of 54.8% was achieved, with mutation carriers exhibiting more severe clinical phenotypes. Notably, the diagnostic rate was significantly higher in younger individuals, underscoring the clinical value of early genetic screening. Beyond traditional Tier 1 gene panels, we identified disease-causing variants in unexpected categories in 4.28% of patients, revealing extensive genotype–phenotype overlap and advocating for a broader diagnostic approach. Some pathogenic variants exhibited normal results in conventional coagulation assays, highlighting the limitations of standard functional testing in detecting underlying genetic causes. These findings establish the ExTH gene panel as a powerful tool for comprehensive genetic diagnosis, capable of capturing clinically relevant variants that would be missed by conventional approaches. This study provides new insight into the molecular landscape of BTPDs, and supports the integration of broad gene panel testing into routine clinical workflows to improve diagnostic accuracy, risk stratification, and personalized treatment.

**Supplementary Information:**

The online version contains supplementary material available at 10.1186/s43556-025-00283-6.

## Introduction

Inherited bleeding, thrombotic, and platelet disorders (BTPDs) are a group of genetic conditions that cause thrombotic, bleeding, or platelet abnormalities [1]. The symptoms of BTPDs are highly heterogeneous, making it difficult to identify the exact cause using standard coagulation tests [2]. This uncertainty in diagnosis often results in clinical misjudgment and the use of less effective treatments. For example, in thrombotic disorders, the initial symptoms are often pulmonary embolism or deep vein thrombosis in the legs [1, 3]. Inherited thrombocytopenia usually presents with mucocutaneous bleeding or nosebleeds. Female patients may also experience heavy menstrual bleeding, and in some cases, other organs such as the heart, bones, or kidneys may be affected [4, 5]. Bleeding caused by coagulation factor deficiencies can range from mild to severe, and some patients may not show any symptoms at all [6, 7]. Due to the complexity of these disorders, patients frequently seek medical care in specialties such as cardiology [8, 9]. This variability in symptoms and the difficulty in diagnosing BTPDs with standard tests create serious clinical problems, frequently resulting in wrong diagnoses and improper treatment.

In recent years, the widespread use of next-generation sequencing (NGS) technology has significantly advanced genetic analysis [10, 11]. This technology enables researchers and clinicians to examine numerous genetic variants in a single test [12]. NGS has played a key role in the development of diagnostic gene panels, which use targeted or exome sequencing to analyze genetic changes associated with inherited BTPDs [1, 4, 13–20]. However, most current studies have focused on specific subtypes of inherited BTPDs, making direct comparisons of diagnostic rates difficult. Additionally, although some large-scale studies, such as those based on the UK Biobank and the Exome Aggregation Consortium (ExAC), have reported BTPDs-related variants in healthy populations, comprehensive screening studies in Asian populations remain limited [21, 22]. This gap restricts our understanding of the prevalence and distribution of BTPDs-associated genetic variants in this demographic, highlighting the need for broader screening efforts.

The rapid advancement of NGS technology has greatly supported the widespread use of genetic panel testing in routine clinical practice [23, 24]. One promising direction is population-based genetic screening, which may help improve early detection of genetic disorders [25, 26]. In light of this, the aim of this study is to develop an NGS-based targeted gene panel that includes the most up-to-date genetic variants linked to inherited BTPDs in the general population. This platform will be assessed for its effectiveness in primary screening, differential diagnosis, and disease staging. The ultimate goal is to improve the overall diagnostic rate of BTPDs and provide a solid basis for making accurate treatment decisions.

## Results

### ExTH gene panel enables high diagnostic yield, especially in younger patients

**Fig. 1:**
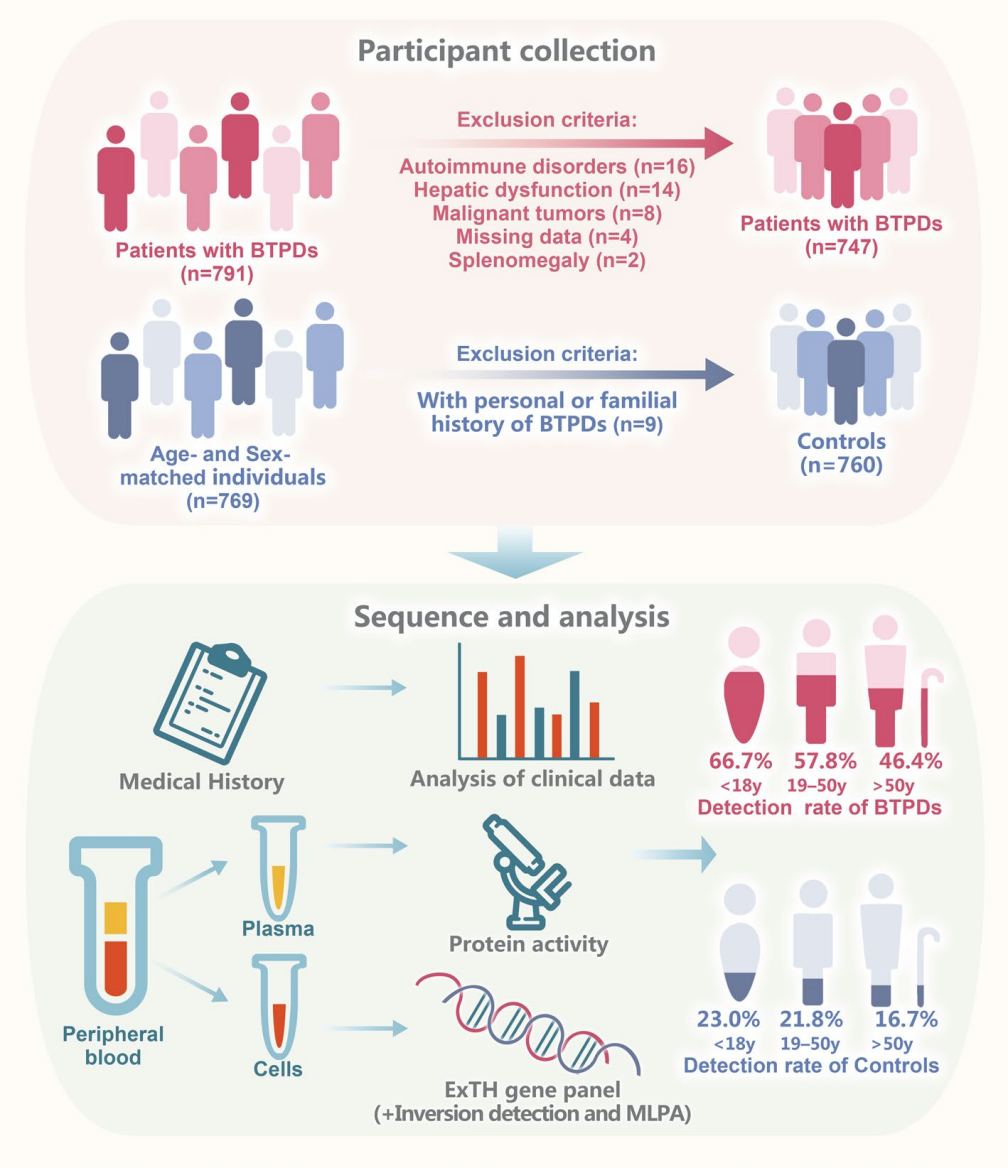
Overview of the study workflow. A total of 747 patients and 760 age- and sex-matched controls were enrolled (detailed inclusion and exclusion criteria are provided). All participants underwent ExTH panel test. The detection rate of patients and controls were subsequently evaluated

**Fig. 2:**
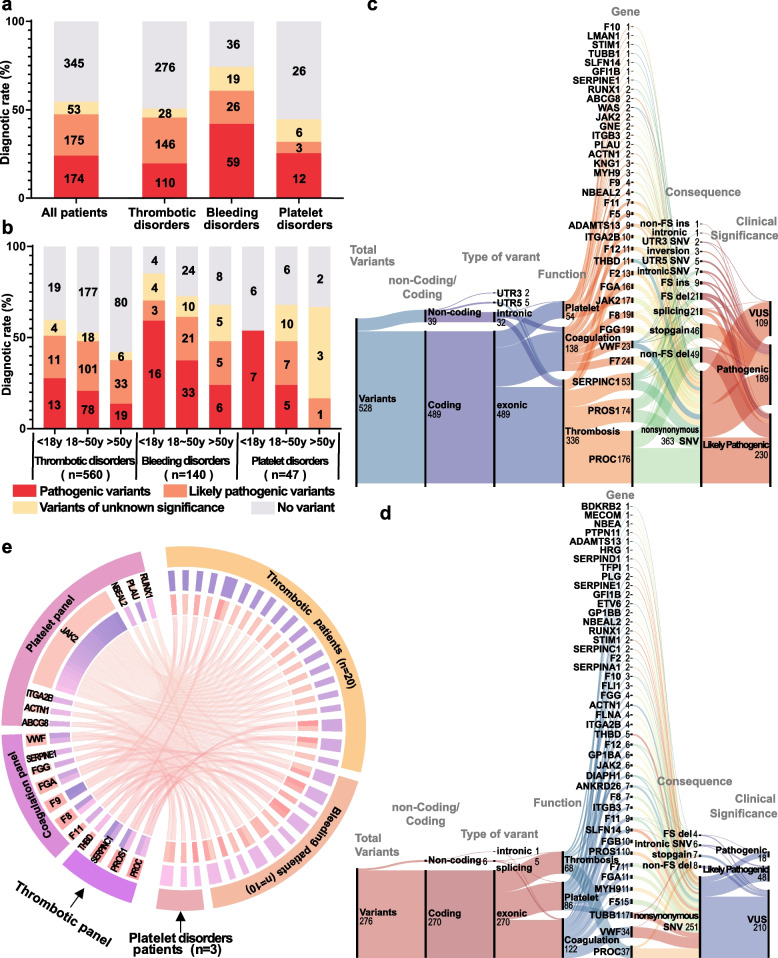
Diagnostic Landscape and Genetic Architecture of BTPDs.** a** Overall diagnostic yield of genetic variants in 747 patients. PVs and LPVs were stratified by disease class: thrombotic (*n* = 560), bleeding (*n* = 140), and platelet disorders (*n* = 47). For patients with multiple variants, only the most clinically significant variant was included. **b** Age-stratified diagnostic yield. Patients were categorized into three groups: pediatric (≤ 18 years, *n* = 87), adult (18–50 years, *n* = 423), and elderly (≥ 50 years, *n* = 237). Diagnostic rates declined with age (Pearson’s *r* = − 0.63, *p* = 0.02). **c** Sankey diagram of disease-associated variants in patients. Flow visualization links clinical phenotypes (left) to specific genetic defects (right), with line thickness proportional to variant frequency. **d** Sankey diagram of variants in 760 controls. Rare variants (MAF < 0.1%) in healthy individuals are mapped to corresponding disease categories. Sankey diagram was generated using RawGraphs (https://www.rawgraphs.io). **e** Circos plot of genotype–phenotype associations. The outer ring (Track 1) represents patients with thrombosis, bleeding, and platelet disorders. The second ring (Track 2) categorizes genes into platelet, coagulation, and thrombosis panels. The third ring (Track 3) and arcs connect patients to the genetic variants they carry, with arc thickness indicating the number of patients with each variant. This diagram highlights the genetic overlap between disease groups and the contribution of specific variants to BTPDs. Abbreviations: FS (frameshift), ins (insertion), del (deletion)

Figure 1 outlines the participant selection and study design workflow. To assess the diagnostic performance of a comprehensive gene panel for detecting genetic mutations in BTPDs, we analyzed 747 index patients (87 children and 660 adults) recruited between January 2019 and January 2022. The cohort consisted of 560 patients with thrombotic disorders, 140 with bleeding disorders, and 47 with platelet disorders. Sequencing achieved an average depth of 650 × and an on-target efficiency of 84.5%. Data processed with GATK (Genome Analysis Toolkit) ensured high quality, with 99.91% of target regions covered at over 30 ×, surpassing clinical-grade standards for reliable variant detection [27]. The overall diagnostic rate of the gene panel was 54.8% (Fig. 2a). Among the 747 patients analyzed, 409 individuals harbored disease-causing mutations, with a total of 531 mutations identified, including 315 distinct mutations. Of these, 32.7% were pathogenic variants (PVs), 37.3% likely pathogenic variants (LPVs), and 29.9% variants of uncertain significance (VUSs) (Table S1). Diagnostic yield varied by BTPD subtype, highest in bleeding disorders (74.3%), followed by thrombotic (50.7%) and platelet disorders (44.7%). An age-dependent decline in diagnostic rate was observed, most notably in thrombotic disorders (59.6% [< 18 years], 52.8% [19–50 years], 42.0% [> 50 years]; *p* = 0.043) and modestly in bleeding disorders (74.1%, 69.3%, 64.0%; *p* = 0.304) (Fig. 2b). Platelet disorders showed variable rates (53.8%, 78.6%, 66.7%; *p* = 0.283), with non-significant trends likely due to the smaller sample size (Table 1). These results suggest higher detection rates in bleeding disorders and earlier genetic diagnosis in younger patients, particularly for thrombotic disorders.

**Table 1:** The diagnostic rates in different age groups

		< 18y		19–50y		> 50y		*p* value
		*N*	Proportion	*N*	Proportion	*N*	Proportion	
Thrombotic (*n* = 560)	with variants	28	59.6%	198	52.8%	58	42.0%	0.043^a^
	without variants	19	40.4%	177	47.2%	80	58.0%	
Bleeding (*n* = 140)	with variants	23	85.2%	64	72.7%	17	68.0%	0.304^a^
	without variants	4	14.8%	24	27.3%	8	32.0%	
Platelet disorders (*n* = 47)	with variants	7	53.8%	22	78.6%	4	66.7%	0.283^b^
	without variants	6	46.2%	6	21.4%	2	33.3%	

*Abbreviations*: *y *years, *N *Number

^a^Based on Pearson's chi-square, ^b^based on Fisher's exact test. *p *< 0.05 was considered statistically significant

Exonic variants were the most common among BTPD patients, with 490 of 531 variants located in exons. Fewer variants were found in introns (*n* = 11), splice sites (*n* = 23), the 5′ untranslated region (UTR) (*n* = 5), and the 3′ UTR (*n* = 2) (Fig. 2c). Thrombosis-related genes (*PROC* and *PROS1*) accounted for 62.6% of mutations (*n* = 336), followed by coagulation-related genes (*n* = 141) and platelet-related genes (*n* = 54). In controls (*n* = 760), coagulation genes had the highest number of variants (*n* = 122), more than platelet-related (*n* = 86) and thrombotic genes (*n* = 68) (Fig. 2d). Notably, 33 patients (4.28%) carried mutations found by gene panels not matching their clinical symptoms (Fig. 2e). This included 20 thrombotic patients with coagulation or platelet gene variants and 7 bleeding patients with thrombotic mutations, highlighting the importance of comprehensive genetic screening rather than restricting testing based on clinical phenotype.

### Mutation spectrum analysis reveals the genetic heterogeneity of BTPDs

**Fig. 3:**
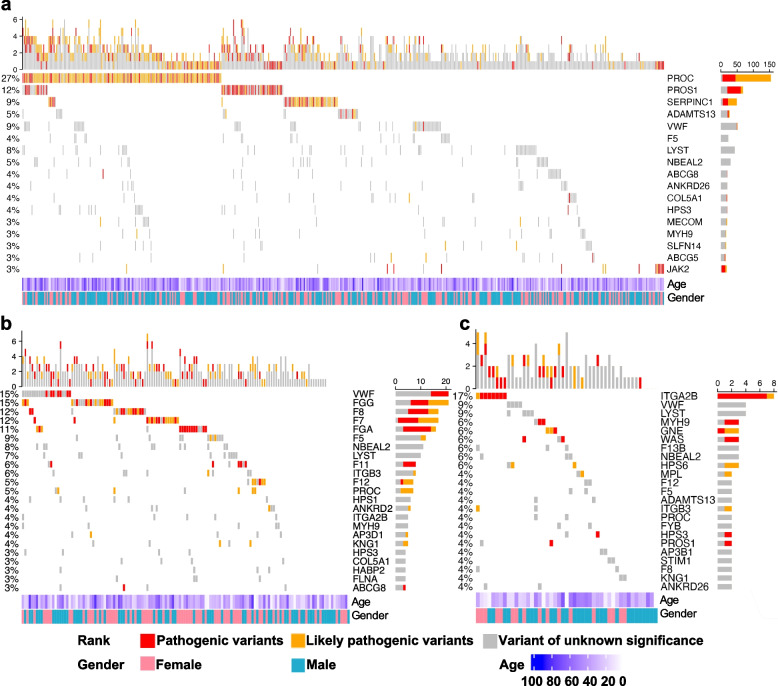
Mutation landscape of bleeding, thrombotic, and platelet disorders. **a** Thrombotic disorders cohort (*n* = 560). Oncoplot displays 284 individuals with pathogenic mutations. **b** Bleeding disorders cohort (*n* = 140). **c** Platelet disorders cohort (*n* = 47). Top panel: Total mutations per sample. Middle panel: Alteration details for genes with mutation frequency ≥ 3%. Bottom panel: Clinical annotations

To systematically identify key genetic contributors to BTPDs, we focused on PVs and LPVs with frequencies above 3% across disease categories. As shown in Fig. 3a, thrombotic disorders commonly involved mutations in *PROC* (27%), *PROS1* (12%), *SERPINC1* (9%), *ADAMTS13* (5%), and *JAK2* (3%). Among bleeding disorders, mutations were primarily found in *VWF* (15%), *FGG* (15%), *F8* (12%), *F7* (12%), and *FGA* (11%) (Fig. 3b). Platelet disorders displayed a different pattern, with *ITGA2B* mutations accounting for 17% of cases, and no other gene exceeding the 3% frequency threshold (Fig. 3c). These results highlight distinct genetic profiles for each BTPD subtype, underscoring the importance of tailored genetic analysis in diagnosis and management.

### Genetic mutations are associated with earlier onset and greater clinical severity in BTPDs

**Fig. 4:**
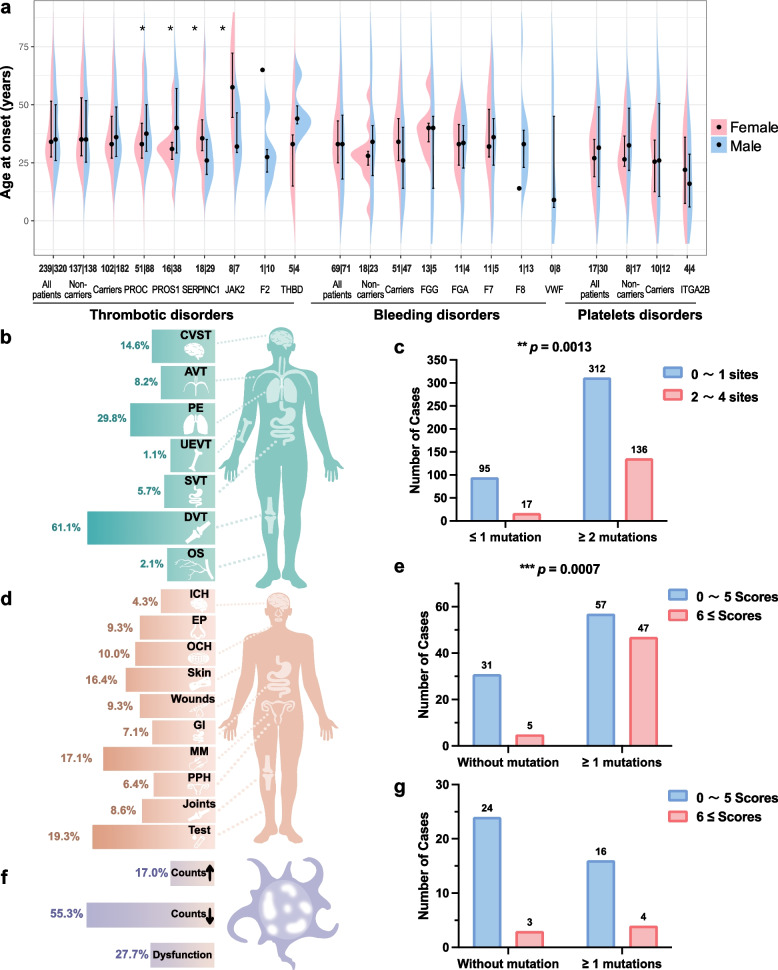
Genotype–phenotype correlations in bleeding, thrombotic, and platelet disorders. **a** Age of onset stratified by mutation status and gender. Kruskal–Wallis tests; *p* < 0.05 (denoted by *). **b** Thrombotic site distribution. **c** Mutation burden vs. thrombotic severity (Chi-square test). **d** Bleeding site distribution. **e** Mutation burden vs. ISTH-BAT scores in bleeding patients (Chi-square test). **f** Platelet disorder subtypes. **g** Mutation burden vs. ISTH-BAT scores in platelet disorders (Chi-square test). Abbreviations: ISTH-BAT (International Society on Thrombosis and Haemostasis Bleeding Assessment Tool), CVST (Cerebral Venous Sinus Thrombosis), AVT (Axial Venous Thrombosis), PE (Pulmonary Thromboembolism), UEVT (Upper Extremity Venous Thrombosis), SVT (Splanchnic Venous Thrombosis), DVT (Deep Venous Thrombosis), and OS (Other Sites), ICH (Intracranial Hemorrhage), EP (Epistaxis), OCH (Oral Cavity Hemorrhage), GI (Gastrointestinal Bleeding), MM (Menorrhagia), and PPH (Postpartum Hemorrhage)

To explore genotype–phenotype correlations, we assessed the impact of mutations on disease severity, focusing on age of onset, thrombotic burden, and bleeding severity. Patients with disease-causing variants tended to have earlier onset, though this was not statistically significant (*p* > 0.05). In thrombotic cases, females with *PROC* or *PROS1* mutations developed symptoms earlier than males, while the reverse was observed for *SERPINC1* and *JAK2* carriers (both *p* < 0.05) (Fig. 4a). Among 560 thrombosis patients, lower extremity thrombosis was most common (61.1%), followed by pulmonary (29.8%) and cerebral venous sinus thrombosis (14.6%) (Fig. 4b). Multi-site thrombosis (27%) was significantly associated with higher mutation burden (*p* = 0.0013; Fig. 4c). In bleeding disorders (*n* = 140), subcutaneous (16.4%) and uterine bleeding (17.1%) were the most frequent symptoms. Severe bleeding was more frequent in mutation carriers (*p* = 0.0013; Fig. 4d–e). Coagulation factor deficiencies were identified in 19.3% of patients, including some asymptomatic cases. Among platelet disorder patients (*n* = 47), 55% had thrombocytosis, 17% thrombocytopenia, and 28% functional platelet defects, with no significant link to mutation status (*p* = 0.34; Fig. 4f–g). These results highlight the clinical importance of genetic testing, especially for identifying patients at risk of multi-site thrombosis and severe bleeding.

### Mutations in *PROC*, *PROS1*, and *SERPINC1* drive anticoagulant deficiency

**Fig. 5:**
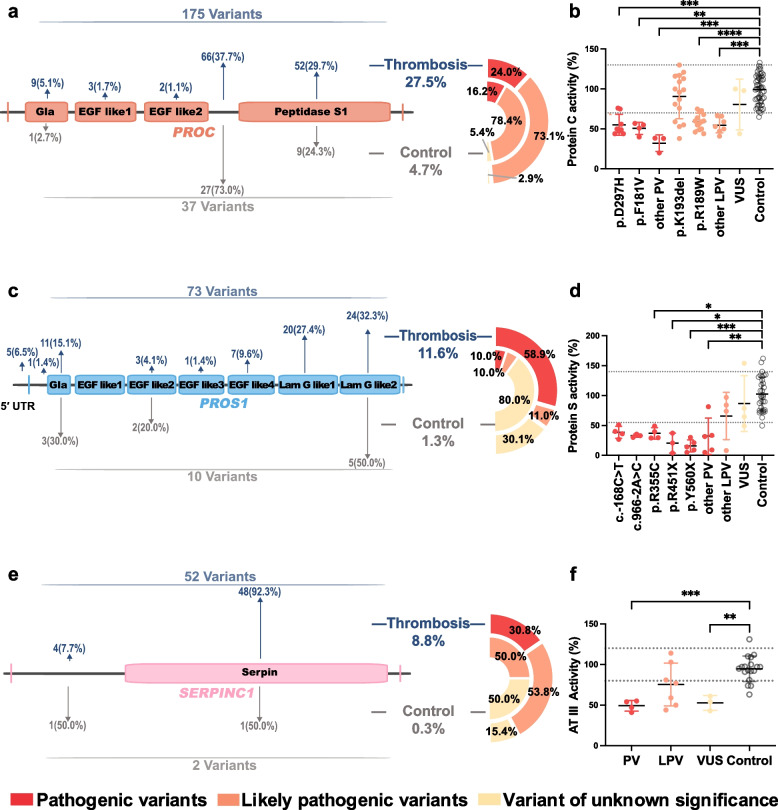
Domain distribution of mutations and their correlation with anticoagulant activity correlations in thrombotic cases. **a** *PROC* mutations in thrombotic cases (*n* = 560) and controls (*n* = 760). PVs, LPVs and VUSs are annotated. **b** PC activity of different mutations, reference range: 70%–130% (dotted lines). **c** *PROS1* mutations. **d** PS activity, reference range: 55%–140% (dotted lines). **e** *SERPINC1* mutations. **f** AT III activity, reference range: 80%–120% (dotted lines). Protein domains defined by UniProt (https://www.uniprot.org) The Kruskal–Wallis test was used in multiple-group comparisons. Control group: individuals without anticoagulant gene mutations

To evaluate the functional impact of genetic mutations, we analyzed protein domain locations and phenotypes of genes with over 20 variants. Among thrombotic patients, *PROC* mutations (*n* = 175 variants, 155 individuals) included 53 distinct types (46 missense, 6 nonsense, 2 nonframeshift deletions, 1 frameshift insertion, and 1 non-coding variant), with 19 novel variants (Table S2, Fig. 5a). Three missense mutations (p.R189 W, p.K193 del, p.D297H) accounted for 61.4% of cases (*n* = 54, 40, and 11, respectively). Most mutations were LPVs clustered in the linker region between the Epidermal Growth Factor-like 2 and Peptidase S1 domains. These mutations were associated with significantly reduced Protein C (PC) activity (*p* < 0.0001), except for p.K193 del (Fig. 5b). In controls (*n* = 760), *PROC* variants were less frequent (4.7%, *n* = 36), with only 7 variants overlapping with patient mutations. A total of 73 variants was identified in 65 patients, including 46 distinct mutations and 20 novel variants. A recurrent hotspot mutation, p.Y560X, was found in 6 cases and localized to the laminin G-like 2 domain (Table S3, Fig. 5c). PVs and LPVs were more common in patients (69.9%) than in controls (1.3%, *n* = 8 variants), and were associated with reduced Protein S (PS) activity (Fig. 5d). *SERPINC1* variants (*n* = 52 in 49 patients) mainly affected the serpin domain. Only two variants (0.3%) were found in controls (Table S4, Fig. 5e–f). In summary, p.R189W, p.K193del, and p.D297H in *PROC*, along with p.Y560X in *PROS1*, were identified as recurrent hotspot mutations among thrombotic patients, highlighting their key roles in thrombosis risk. Notably, some pathogenic variants showed normal activity in standard coagulation tests, revealing the limitations of conventional functional assays in accurately diagnosing BTPDs.

### Mutations in *FGA*, *FGG*, and *F8* impair factor levels and contribute to bleeding severity

**Fig. 6:**
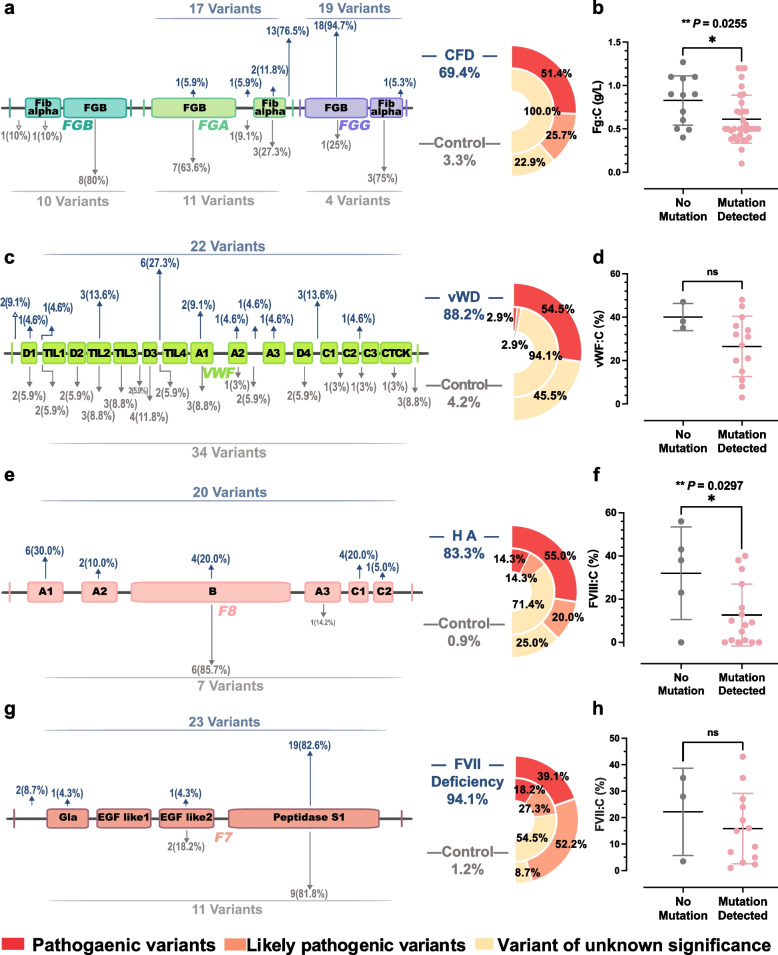
Domain distribution of mutations and their correlation with coagulant activity in bleeding disorders. **a** *FGA*, *FGB*, and *FGG* mutations in bleeding cases (*n* = 140) and controls (*n* = 760). **b** Fibrinogen activity (Fg:C, g/L). **c** *VWF* mutations. **d** von Willebrand factor activity (vWF:C, %). **e** *F8* mutations. **f** Factor VIII activity (FVIII:C, g/L). **g** *F7* mutations. Protein domains defined by UniProt (https://www.uniprot.org). **h** Factor VII activity (FVII:C, g/L). Student’s t test was used. Control group: individuals without coagulation-related gene mutations

To evaluate the effects of gene mutations on coagulation factor activity, we analyzed mutation distribution and associated phenotypes in genes with ≥ 20 mutations. Among fibrinogen (Fg) deficiency patients (*n* = 49), mutations were found in 69.4% (*n* = 34), mainly in *FGG* and *FGA* (Fig. 6a, Table S5). The recurrent mutation *FGA*:p.R35H (*n* = 8) affected *α*-chain polymerization domains, and carriers had significantly lower Fg levels than non-carriers (*p* = 0.0255; Fig. 6b). In von Willebrand disease (VWD), 88.2% of patients had *VWF* mutations, including splice-site and nonsense variants linked to type 2 VWD, though vWF activity was not significantly different between groups (Fig. 6c–d, Table S6). In FVIII deficiency, *F8* mutations were identified in 76.9% of patients, primarily clustered in the A1 domain. Severe variants caused FVIII:C < 1%, while mild variants retained 5%–38% activity. Mutation carriers had significantly lower FVIII:C levels than non-carriers (*p* = 0.0297; Fig. 6e–f, Table S7). In FVII deficiency (*n* = 17), 94.1% carried *F7* mutations, primarily affecting the Peptidase S1 domain, though FVII:C levels did not differ significantly between carriers and non-carriers (Fig. 6g–h, Table S8). Overall, Fg, vWF, FVIII, and FVII deficiencies were the most common bleeding phenotypes. Mutations of *FGA*, *FGG* and *F8* were significantly associated with reduced factor levels. Most variants in controls were classified as VUSs with no clear phenotypic impact.

### *HPS6* and *MTHFR* contribute to the polygenic architecture of hereditary thrombophilia

**Fig. 7:**
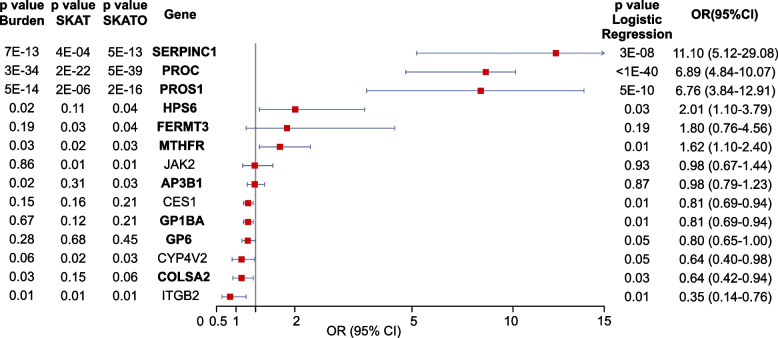
Genome-wide association analysis of thrombophilia-related genes. The forest plot illustrates effect sizes (OR) and 95% CI for Burden, SKAT and SKAT-O analyses. Genes are categorized as Panel I (diagnostic-grade genes, bold font) and Panel II (candidate susceptibility genes, regular font). The burden, SKAT/SKAT-O test and logistic regression were used. Only associations with *p* < 0.05 are displayed

To explore genetic associations with thrombosis in the Asian population, we conducted a gene-based association analysis involving 560 thrombotic patients and 760 controls. We used Burden tests, the Sequence Kernel Association Test (SKAT), SKAT with Optimal weights (SKAT-O), and logistic regression, focusing on coding variants with a minor allele frequency (MAF) below 5%. The analysis identified nominal associations (*p* < 0.05) in 14 genes, with four candidate susceptibility genes highlighted (Fig. 7). Among these, *SERPINC1*, *PROC*, and *PROS1* showed significant genome-wide associations and high Odds Ratios (OR): *SERPINC1* (OR = 11.10, 95% CI: 5.12–29.08), *PROC* (OR = 6.89, 95% CI: 4.84–10.07), and *PROS1* (OR = 6.76, 95% CI: 3.84–12.91), indicating a strong genetic influence on thrombotic risk. These findings emphasize *SERPINC1*, *PROC*, and *PROS1* as major contributors to thrombosis risk, while *HPS6* and *MTHFR* may have smaller but meaningful effects, supporting the idea that hereditary thrombophilia is polygenic.

## Discussion

This study represents the largest investigation to date of NGS-based multigene panel testing for hereditary BTPDs in an Asian population. Our results demonstrate that comprehensive gene panels substantially improve diagnostic sensitivity, particularly in younger individuals, that might be missed with conventional single-gene or Tier 1 testing. This reinforces the clinical value of broad NGS panels in early diagnosis and personalized patient management.

Current diagnostic strategies often rely on phenotype-driven Tier 1 gene testing to reduce costs and workload, but our findings highlight the limitations of this approach [28–30]. Using a comprehensive panel, we identified actionable variants in 4.28% of patients from non-phenotype-matched genes, underscoring the overlapping mechanisms in hemostatic pathways [6, 31, 32]. For example, mutations in *PROC*, which regulate activated Factors V and VIII, can influence thrombosis even when PC activity appears normal [33]. We found two unrelated children with thrombosis were found to carry the hemizygous *F9* p.R346Q mutation, with elevated FIX activity (200% and 231%) and thrombi in major veins. This variant, previously reported as a VUS in the 1990 s due to technological limitations, affects the same residue as p.R346L, which was linked to markedly increased FIX activity (776%) in a thrombophilic Italian male [34, 35]. In contrast, a different substitution at a nearby site (p.R338P) causes Hemophilia B [36]. These findings suggest that p.R346Q and p.R346L are gain-of-function mutations contributing to thrombophilia, highlighting the value of comprehensive gene panels in identifying clinically relevant variants.

The enhanced diagnostic yield among younger patients in our cohort has important implications. Inherited BTPDs typically manifest earlier in life, and genetic factors play a dominant role in pediatric cases [37]. In contrast, environmental factors (e.g., aging, immobility, and medications) increasingly influence disease risk with age [31]. Thus, NGS-based genetic testing is particularly valuable for early detection and intervention in younger populations, aligning with current understanding of gene-environment interactions in hemostasis [38].

Structural domain mapping also revealed that many pathogenic variants cluster in functionally critical regions. For instance, *FGA* mutations were enriched in the alpha C-terminal polymerization domain, while *F8* mutations primarily affected the A1 domain essential for vWF binding [39, 40]. Mutations in FVII deficiency were mostly found in the Peptidase S1 domain, which is vital for the activation of Factor X in the coagulation cascade [41]. These observations highlight the structural basis of pathogenicity and support the use of genetic profiling to guide precision therapies.

Our data also provide insights into the mutation spectrum in the Chinese population. Thrombophilia-associated variants common in Caucasians, such as *F5* Leiden and *F2* G20210 A, were absent in our cohort, consistent with previous studies [42]. Several variants, such as *PROC*:p.R189 W and p.K193 del, were more prevalent in our population than reported in East Asian datasets from gnomAD, suggesting a potential population-specific variation. Our control group analysis further revealed a substantial carrier burden of PVs and LPVs (7.3% in thrombotic disorders, 2.1% in bleeding disorders, and 8.8% in platelet disorders), with 76.1% of variants classified as VUSs. Notably, 39.8% of the variants occurred in recessive genes, which may have clinical implications for reproductive counseling and family screening [43, 44]. Although biallelic inheritance in recessive genes is rare, carriers of PVs and LPVs still pose a genetic risk to offspring, especially if both parents carry the same mutation [45, 46]. This highlights the relevance of these variants in the general population for reproductive planning and genetic counseling [47].

This study has several limitations. Despite a relatively large sample size, data were collected from a single center, potentially limiting generalizability. Clinical data, particularly treatment responses, were incomplete, hindering assessment of genotype–phenotype correlations. Additionally, we did not perform functional validation, so the pathogenicity of some variants remains uncertain. These issues underscore the need for multicenter studies and functional assays to strengthen clinical applicability. Encouragingly, the declining cost of NGS (now < $10 per genome) may soon enable broader genomic profiling, larger cohorts, and integration of multi-omics data, improving statistical power and phenotype**–**genotype correlation [48]. Such advances will enhance the diagnostic and prognostic value of genetic testing for BTPDs.

## Conclusion

This study shows that comprehensive NGS panels enhance BTPD diagnosis, especially in younger patients, by detecting a broader range of pathogenic variants missed by Tier 1 tests. The findings support incorporating broad genetic testing into clinical practice to improve diagnosis, treatment, and reproductive counseling.

## Materials and methods

### Patients and controls

From January 2019 to January 2022, patients diagnosed with BTPDs were recruited at Union Hospital, Tongji Medical College, Huazhong University of Science and Technology, Wuhan, China. The exclusion criteria included a previous or current diagnosis of hepatic disease, malignancies, splenomegaly, and autoimmune disease. The sample collection was previously described [49]. Family history was confirmed through personal interviews and review of medical records. The ExTH gene panel was initially performed in 791 patients. After applying inclusion criteria, 44 cases were excluded from the final analysis. The exclusion criteria comprised: autoimmune disorders (*n* = 16), hepatic dysfunction (*n* = 14), malignant neoplasms (*n* = 8), splenomegaly (*n* = 2), and cases with incomplete clinical data (*n* = 4). Age- and sex-matched control participants were recruited during the same period. 9 individuals were excluded due to a reported personal or family history of BTPDs. All participants in this study were ethnically Han Chinese. Ultimately, a total of 747 cases and 760 controls were available for the statistical evaluation. This study was approved by the ethics committee of Union Hospital, Huazhong University of Science and Technology ([2022] Ethics Review Serial Number (0157)), and complied with the principles expressed in the Declaration of Helsinki. Prior to study enrollment, written informed consent was obtained from all participants in accordance with institutional ethical guidelines.

### Sequencing procedure

This panel was designed to capture all protein-coding regions and 10 bp of flanking intronic sequences across 130 genes, covering most genes associated with BTPDs. The ExTH gene panel included two components: (I) diagnostic-grade genes, comprising (I a) a thrombotic panel, (I b) a coagulation panel, and (I c) a platelet disorder panel; and (II) candidate susceptibility genes (Table S9).

Genomic DNA was extracted from peripheral blood using the QIAamp DNA Blood Kit (Qiagen, Germany). Library preparation was performed using the IGT® Enzyme Plus Library Prep Kit (iGeneTech Bioscience, China), followed by target enrichment with the IGT® TargetSeq Kit (iGeneTech Bioscience, China). Sequencing was carried out on the Illumina HiSeq 4000 platform (Illumina, USA). Raw sequencing data were aligned to the human reference genome (hg19) using the BWA-MEM algorithm. Annotation was performed using multiple databases, including Ensembl Genome Database, Consensus Coding Sequence Project (CCDS), GENCODE Project, Vertebrate Genome Annotation (VEGA), Single Nucleotide Polymorphism (SNP) Database, and Cytogenetic Band (CytoBand) databases. Data quality metrics, such as alignment rate, duplication rate, and base quality, were assessed using samtools stats (v1.9). Sequencing depth across target regions was calculated with samtools depth.

For patients with confirmed coagulation factor deficiencies but negative results on the ExTH gene panel, additional genetic testing was performed. This included inversion detection and multiplex ligation-dependent probe amplification (MLPA) targeting the relevant gene (Supplementary Methods A and B). Confirmed variants were validated by Sanger sequencing in both the affected individuals and their family members [50, 51].

### Data processing

We used the Human Gene Mutation Database (HGMD) and the Clinical Variant Database to determine whether a mutation was novel. Genetic interpretation was conducted following the guidelines of the American College of Medical Genetics and Genomics (ACMG) and the Association for Molecular Pathology (AMP) to classify variants as PVs, LPVs, VUSs or (likely) benign variants [27, 52]. For disease-specific annotation, we referred to curated resources such as the European Association for Haemophilia and Allied Disorders (EAHAD) database (http://www.eahad-db.org/), the Leiden Open Variation Database (LOVD) (https://www.lovd.nl/), and the Glanzmann Thrombasthenia Database (https://glanzmann.mcw.edu/) [53]. Additionally, we used previously reported variants from ThromboGenomics (http://www.thrombogenomics.org.uk/) for prioritization. To integrate genetic findings with clinical and laboratory data, multidisciplinary team (MDT) meetings were held, involving clinicians, geneticists, and laboratory specialists [54]. Variants in recessive BTPDs were reported only when present in the homozygous or compound heterozygous state. Given the complexity of interpreting noncoding variants, we focused on intronic and regulatory variants previously reported to be associated with the disease.

### Coagulation factor activity testing and ISTH-BAT scores

The activities of PC, PS, AT, vWF, Fg, and coagulation Factor VII, VIII, IX, X, XI, and XII were measured using validated commercial assays. Details of the reagents used are listed in Table S10. Factor XIII activity was assessed by the urea solubility test, as described in Supplementary Methods C. All coagulation assays were performed on a STA® automated coagulation analyzer (Diagnostica Stago, Asnières-sur-Seine, France). To avoid interference from anticoagulant therapy, test results obtained within two weeks of anticoagulant use were excluded from analysis [52, 55]. Coagulation screening in the population group was performed using the same assays as in the patient group. Bleeding phenotypes were evaluated using the ISTH-BAT scoring system [56].

### Statistical analysis

Data were computerized and analyzed using GraphPad Prism 9.5.0 and R statistical analysis software 4.3.0. For datasets fulfilling normality and homogeneity, group differences were evaluated with two-tailed Student's t-tests. Non-normally distributed data were analyzed via Kruskal–Wallis tests. Comparing proportions was done using Chi-square and Fisher's exact tests.

We performed gene-based association analyses focusing on rare coding variants—including missense, splice site, nonsense, and frameshift mutations—with a minor allele frequency (MAF) of less than 5% [57]. For each gene, the number of rare variant alleles carried by each individual was calculated, and this count was used as the independent variable in logistic regression analysis [58]. The cumulative burden of these variants in cases and controls was then evaluated using the Burden test, SKAT, and SKAT-O, implemented via the SKAT R package [59]. The OR, CI and *p* was calculated to assess the association between genetic variants and thrombotic events adjusting for age and sex. *p* = 1.34 × 10^−6^ (0.05/18666/2) was the threshold for significance according to the number of genes in CCDS Release 15 [60].

## Supplementary Information

Supplementary Material 1.

Supplementary Material 2.

## Data Availability

All data generated or analyzed in this study are available within this published article. The main data supporting the findings of this study are included in the main text and Supplementary Materials. Additional raw data are not openly available due to reasons of sensitivity but can be obtained from the corresponding author upon reasonable request.
