# Supplementary material for: Application of multigene panel testing for bleeding, thrombotic, and platelet disorders in patients and the general population in China
Source: Mol Biomed. 2025 Jun 9;6:39. doi: 10.1186/s43556-025-00283-6 (PMC12149383; doi:10.1186/s43556-025-00283-6)
Supplement: Supplementary file 2 — Supplementary Material 2. [file 43556_2025_283_MOESM2_ESM.docx]

**Application of Multigene Panel Testing for Bleeding, Thrombotic, and Platelet Disorders in Patients and the General Population in China**

**Short running title:** Hemostatic Disorder Genetics Study in China

**Authors' information：**

Yaohua Cai, PhD ^1,2¶^; Wenyi Lin, PhD ^1,2¶^; Jun Deng, PhD ^1,2,3¶^; Zhipeng Cheng, PhD ^1,2,3^; Yanyi Tao, PhD ^1,2^; Hui Lu, PhD ^1,2^; Yunqing Xia, PhD ^1,2^; Tingting Wu, PhD ^1,2^; Liang V. Tang, PhD ^1,2,3*^; Yu Hu, PhD ^1,2,3*^

1 Institute of Hematology, Union Hospital, Tongji Medical College, Huazhong University of Science and Technology, Wuhan, Hubei 430022, China.

2 Key Laboratory of Biological Targeted Therapy (Huazhong University of Science and Technology), Ministry of Education, Wuhan, Hubei 430022, China.

3 Hubei Clinical and Research Center of Thrombosis and Hemostasis, Wuhan, Hubei 430022, China.

¶ Yaohua Cai, Wenyi Lin and Jun Deng contributed equally to this study.

* Corresponding author:

Liang V. Tang and Yu Hu, PhD. (E-mail: lancet.tang@qq.com and dr_huyu@126.com)

Institute of Hematology, Union Hospital, Tongji Medical College, Huazhong University of Science and Technology.

No. 1277 Jiefang Avenue, Wuhan, Hubei 430022, China.

Tel.: +86 27 85726335, Fax: +86 27 85726387.

**Supplementary Methods**

**I. Supplementary Methods A. Detection of F8 Intron 22 Inversion and Intron 1 Inversion**

**II. Supplementary Methods B. Multiplex Ligation-dependent Probe Amplification (MLPA) for Large Deletions and Duplications**

**III. Supplementary Methods C. Urea Solubility Test for Factor XIII Activity**

**Supplementary Methods A.**

**Detection of F8 Intron 22 Inversion and Intron 1 Inversion**

Long-distance Polymerase Chain Reaction (LD-PCR) was performed to detect intron 22 inversions in the F8 gene, according to the 2005 guidelines established by the UK Haemophilia Centre Doctors' Organisation (UKHCDO)[1]. Each 25 μL PCR reaction contained 12.5 μL of 2× GC Buffer I, 4 μL of dNTPs (with a dGTP to 7-deaza-dGTP ratio of 3:1), 1 μL of primer P (10 μmol/L), 0.5 μL each of primers Q and B (10 μmol/L), 1 μL of genomic DNA (~100 ng), 0.25 μL of LA Taq polymerase (TaKaRa), and nuclease-free water to final volume.The thermal cycling protocol was as follows: initial denaturation at 98°C for 1 min; 10 cycles of 98°C for 10 s and 68°C for 10 min; followed by 20 cycles of 98°C for 10 s and 68°C for 20 s; and a final extension at 72°C for 10 min.PCR products were separated by agarose gel electrophoresis in 1× TBE buffer and visualized using a gel documentation system.

The following primers were used (expected amplicon sizes were 11 kb or 12 kb):

Primer P: 5′-TGCCTGTCCATTACACTGATGACATTATGCTGAC-3′

Primer Q: 5′-TACAACCATTCTGCCTTTCACTTTCAGTGCAATA-3′

Primer B: 5′-CCAAACTATAACCAGCACCTTGAACTTCCCCTCTCA-3′

Intron 1 inversion of the F8 gene was analyzed using two duplex PCR assays, Int1h-1 and Int1h-2, as previously described[1]. Expected amplicon sizes were 1776 bp or 1991 bp, depending on inversion status. Each 25 μL PCR reaction contained 2.5 μL of 10× LA Buffer II (with Mg²⁺), 2 μL of dNTP mix, 1 μL of each primer (10 μmol/L), 2 μL of genomic DNA (~200 ng), 0.25 μL of LA Taq polymerase, and nuclease-free water to volume. PCR was performed under the following conditions: initial denaturation at 94°C for 5 min; 30 cycles of 94°C for 10 s, 61°C for 30 s, and 72°C for 2 min; followed by a final extension at 72°C for 10 min. PCR products were analyzed by agarose gel electrophoresis in 0.5× TBE buffer and visualized under UV illumination.

For the Int1h-1 reaction, the following primers were used (expected amplicon sizes were 1323 bp or 1908 bp):

Primer 9F: 5′-GTTGTTGGGAATGGTTACGG-3′

Primer Int1h-2F: 5′-GGCAGGGATCTTGTTGGTAAA-3′

Primer 9CR: 5′-CTAGCTTGAGCTCCCTGTGG-3′

For the Int1h-2 reaction, the primers were (expected amplicon sizes were 1776 bp or 1991 bp):

Primer 9F: 5′-GTTGTTGGGAATGGTTACGG-3′

Primer Int1h-2F: 5′-GGCAGGGATCTTGTTGGTAAA-3′

Primer Int1h-2R: 5′-TGGGTGATATAAGCTGCTGAGCTA-3′

**Supplementary Methods B.**

**Multiplex Ligation-dependent Probe Amplification (MLPA) for Large Deletions and Duplications**

For samples negative by both high-throughput sequencing and inversion analysis, Multiplex Ligation-dependent Probe Amplification (MLPA) was employed to detect large deletions and duplications. MLPA was performed using commercially available kits (SALSA MLPA P011-B1 and P012-B1, MRC-Holland, Amsterdam, The Netherlands) in accordance with the manufacturer's instructions.

**Supplementary Methods C.**

**Urea Solubility Test for Factor XIII Activity**

The urea solubility test was used to assess Factor XIII (FXIII) activity by evaluating the stability of a fibrin clot[2]. A clot is formed in a plasma sample using calcium chloride. The clot is then incubated in a 5M urea solution for 24 hours. A normal clot will resist dissolution, indicating functional Factor XIII activity. If the clot dissolves, it suggests a deficiency or dysfunction of Factor XIII.

**References**

1. Mitchell M, Keeney S, Goodeve A. The molecular analysis of haemophilia B: a guideline from the UK haemophilia centre doctors' organization haemophilia genetics laboratory network. Haemophilia. 2005;11(4):398-404. doi:10.1111/j.1365-2516.2005.01112.x.

2. Dorgalaleh A, Tabibian S, Assadollahi V, Shamsizadeh M, Zareban I, Soori S et al. Comparison of 2 Methods of Clot Solubility Testing in Detection of Factor XIII Deficiency. Lab Med. 2016;47(4):283-5. doi:10.1093/labmed/lmw046.
